# N, S-Doped Carbon Dots Prepared by Peanut Protein Isolates and Cysteamine as Highly Sensitive Fluorescent Sensors for Fe^2+^, Fe^3+^ and Lactoferrin

**DOI:** 10.3390/polym15010216

**Published:** 2022-12-31

**Authors:** Xinxin Wang, Xinyu Zu, Ting Wang, Yanan Zhao, Yan Liang, Xiaochen Wang, Qian Chai, Yunjuan Zhang, Hongzhong Chen, Hua Wang

**Affiliations:** 1 State Key Laboratory of Biobased Material and Green Papermaking, School of Food Sciences and Engineering, Qilu University of Technology (Shandong Academy of Sciences), Jinan 250353, China; 2 College of Biotechnology, Qilu University of Technology (Shandong Academy of Sciences), Jinan 250353, China; 3 Shandong Center for Food and Drug Evaluation & Inspection, Jinan 250014, China; 4 School of Materials Science and Engineering, Shandong University, Jinan 250061, China

**Keywords:** N, S-PPI-CDs, fluorescence probe, biocompatibility, Fe^2+^/Fe^3+^ detection, lactoferrin supplement detection

## Abstract

Lactoferrin (LF) is an iron-binding glycoprotein with various biological activities that has been extensively used in food and medical applications. Several methods for detecting LF have been reported, but they still face challenges in terms of sensitivity and simplicity of detection. To achieve an accurate and efficient detection of LF, we developed a method for the determination of LF in lactoferrin supplements using carbon dots (CDs) fluorescent probes. The N, S-doped PPI carbon dots (N, S-PPI-CDs) were prepared using a protein (peanut protein isolate) and cysteamine as precursors. The prepared N, S-PPI-CDs exhibited intense blue fluorescence and good biocompatibility, while the fluorescence intensity of the N, S-PPI-CDs showed a good linear relationship with Fe^2+^/Fe^3+^ concentration (0–2 μM). The N, S-PPI-CDs exhibited a high potential ability to rapidly detect Fe^2+^/Fe^3+^ within 30 s, with a limit of detection (LoD) of 0.21 μM/0.17 μM. Due to the reversible binding of LF to Fe, the N, S-PPI-CDs showed a high sensitivity and selectivity for LF, with a limit of detection (LoD) of 1.92 μg/mL. In addition, LF was quantified in real sample LF supplements and showed a fluctuation in recovery of less than 2.48%, further demonstrating the effectiveness of the fluorescent N, S-PPI-CDs sensor.

## 1. Introduction

Lactoferrin (LF) is an iron-binding functional glycoprotein in the transferrin family with a molecular weight of 80 kDa [1], which is widely distributed in human and mammalian milk, other tissues and their secretions [1,2]. LF is generally considered as the first natural immune barrier to protect the body from microbial infection [3]. In addition, it exhibits various biological activity, including antibacterial activities, antiviral activities, anti-inflammatory activities, anticancer activities, antioxidant activities and enzyme catalytic activities [4,5,6,7]. To date, LF has been widely used in cosmetics, food, animal production, medical treatment and other fields [8]. However, the structure of LF is easily disrupted during the production process, which may result in the LF content of a final product not reaching the added level or the initial level [9]. Therefore, a reliable LF detection method urgently needs to be established to ensure the quality of products. A variety of determination methods for LF have been developed, including reversed-phase high-performance liquid chromatography (RP-HPLC) [10], liquid chromatography–tandem mass spectrometry (LC−MS/MS) [11], the enzyme-linked immunosorbent assay (ELISA) [12], the surface plasmon resonance (SPR)-based immunosensor [13], radial immunodiffusion (RID) [14] and capillary electrophoresis (CE) [15]. However, each of these analytical methods has its disadvantages. For instance, RP-HPLC is regarded as a time-saving and high-accuracy detection method, but it has poor selectivity for low LF content [1]. In contrast, LC−MS/MS can achieve the highly sensitive and selective detection of LF, but its sample pretreatment is time-consuming [16]. Similarly, ELISA also has high sensitivity and selectivity for LF, while it has the disadvantages of low efficiency and high cost [3]. The surface plasmon resonance (SPR)-based immunosensor is considered as a real-time and automated method, but it is easily affected by temperature and sample composition [11]. The RID method has high specificity analysis but low accuracy [1]. CE shows the advantages of lower sample consumption and high precision, but its poor reproducibility greatly limits the practical application [1,3]. Hence, an efficient and accurate technique for LF quantification is highly needed.

LF has a polypeptide chain with about 690 amino acid residues and forms a basically symmetrical bilobal structure, while each lobe can reversibly bind to Fe^2+^/Fe^3+^ [1,17,18]. It was reported that iron binding can obviously affect the biological functions of LF. For example, the chelation of LF with irons can competitively inhibit the growth of bacteria, thus endowing LF with antibacterial activity [19]. Furthermore, Gibbons’ s group reported that LF iron saturation levels can affect its anti-tumor activity, apoptosis and cytotoxicity [20]. Meanwhile, binding irons will increase the stability of LF, which makes its structure stronger [21]. On the other hand, the combination of LF and irons can effectively promote the iron absorption of the intestine [22]. The above results indicate that LF and iron complement each other to a certain extent. Therefore, the bioactivity of LF can be analyzed by the quantitative detection of iron in LF.

As a new kind of carbon-based fluorescent nanomaterial, carbon dots have already been widely used in chemical sensing and biosensing due to their good biocompatibility, excellent optical properties and chemical stability and are emerging in the detection of active substances [23]. Han et al. reported nitrogen-doped carbon dots with high selectivity toward apoferritin in aqueous medium, which was also successfully applied in fluorescence imaging of living cells [24]. Wang et al. obtained a “turn-on-off-on” fluorescence switch based on quantum dots and gold nanoparticles, which was utilized in ovotransferrin detection of egg powder [25]. Zhang et al. reported a new dual-excitation and dual-emission fluorescent probe based on carbon quantum dots for the detection of cysteine, homocysteine, glutathione and hydrogen sulfide in living cells [26]. However, fluorescent detection on LF with carbon dots has been rarely reported so far.

**Scheme 1:**
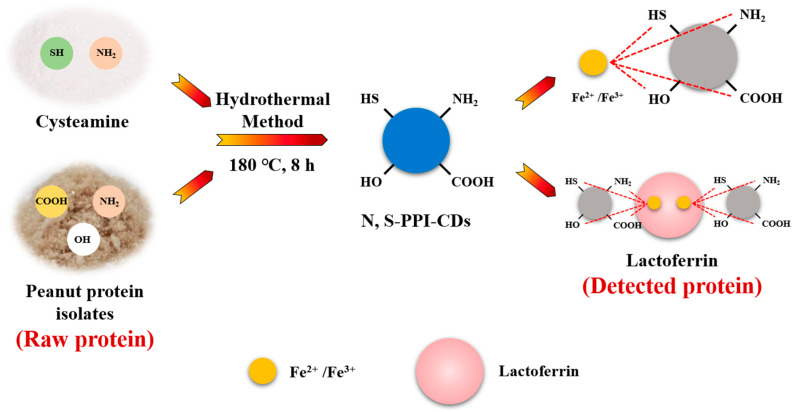
The preparation and application of N, S-PPI-CDs.

It has been reported that N, S co-doped carbon dots have good luminescence performance and stability. Moreover, the introduction of N and S can effectively improve the sensitivity and selectivity of carbon dots as fluorescent probes [27,28]. Peanut protein isolates (PPI) are rich in hydroxyl, amino and carboxyl groups, while cysteamine is rich in amino and sulfhydryl groups. Herein, we used PPI and cysteamine as precursors to prepare the N, S co-doped carbon dots by the hydrothermal method (Scheme 1). This process was conducive to improving the N content of carbon dots and introducing S. Thus, the superiority of carbon dots as fluorescent sensors is further enhanced. The structure, composition and morphology of the N, S-PPI-CDs were studied by Fourier-transform infrared (FTIR) spectra, X-ray photoelectron spectra, atomic force microscopy (AFM) images and transmission electron microscope (TEM) images. The spectral characteristics of the N, S-PPI-CDs were characterized by ultraviolet (UV) absorption spectra and photoluminescence (PL) spectra. Furthermore, the biocompatibility of the N, S-PPI-CDs was assessed by a toxicity test. A detailed study was conducted on the FL interactions between the N, S-PPI-CDs and Fe^2+^/Fe^3+^ and LF. Moreover, the application of this proposed method to real LF samples was verified.

## 2. Materials and Methods

### 2.1. Materials

Cysteamine and inorganic salts (including FeCl_2_·4H_2_O, FeCl_3_·6H_2_O, CaCl_2_·2H_2_O, CoCl_2_·6H_2_O, CrCl_3_·6H_2_O, CuCl_2_·2H_2_O, HgCl_2_, MgCl_2_·6H_2_O, MnCl_2_·4H_2_O, PbCl_2_ and ZnCl_2_) were obtained from Aladdin Bio-Chem Technology Co., Ltd. (Shanghai, China). Lactoferrin (iron content ≤ 0.209 g/g) derived from bovine milk was acquired from Macklin Biochemical Co., Ltd. (Shanghai, China). Peanut protein isolates (PPI) were obtained from low-temperature defatted peanut meal by an alkali extraction and acid precipitation method. *Lactobacillus plantarum* strain was cultivated by our laboratory. Lactoferrin supplement drops were purchased from local market.

### 2.2. Characterization

The FTIR spectra were measured by utilizing an FTIR spectrometer (Thermo Scientific Nicolet iS10, Thermo Fisher Scientific, Shanghai, China). UV absorption spectra were collected from UV-9 000 spectrophotometer (Metash, Shanghai, China). PL spectra were obtained from F2700 Fluorescence spectrophotometer (Hitachi, Tokyo, Japan). Multimode8 Atomic force scanning probe microscope (Bruker, Beijing, China) was used to obtain AFM images. A transmission electron microscope (FEI Tecnai G2 F20, FEI, Shanghai, China) was applied to obtain TEM images. X-ray photoelectron spectroscopy (XPS) was acquired by using an X-ray photoelectron spectrometer (Thermo Fisher ESCALAB Xi+, Thermo Fisher Scientific, Shanghai, China).

### 2.3. Synthesis of PPI-CDs and N, S-PPI-CDs

PPI-CDs and N, S-PPI-CDs were prepared by hydrothermal method. Firstly, PPI (0.3 g) was dissolved in 10 mL distilled water and transferred into a Teflon reactor that was heated at 180 °C for 8 h. The obtained carbon dot solution was filtered through 0.22 µm membrane filters and then freeze-dried to acquire PPI-CDs. Similarly, by the above procedure, N, S-PPI-CDs were prepared from PPI (0.3 g) and cysteamine (0.3 g). The prepared PPI-CDs and N, S-PPI-CDs were stored in dryer until further testing.

### 2.4. Biocompatibility of PPI-CDs and N, S-PPI-CDs

To evaluate the biocompatibilities of PPI-CDs and N, S-PPI-CDs, toxicity experiments were carried out. Given its application in LF assay, *Lactobacillus plantarum* was chosen as a model, and its survival rate was used as a toxicity indicator. The control group was obtained by inoculating *Lactobacillus plantarum* into MRS liquid medium at 1% inoculum and incubating continuously at 37 °C for 24 h. Meanwhile, 1 g/L of PPI-CDs or N, S-PPI-CDs solution was used to partially replace H_2_O (50%, 70% and 90%) or Glucose (GLU) (10%, 20% and 30%). Then, the bacterial solution was diluted according to the gradient and inoculated on the MRS solid medium to prepare reference sample and testing sample. The toxicity of PPI-CDs or N, S-PPI-CDs was investigated by the viable count.

### 2.5. Fluorescence Selectivity and Interference of N, S-PPI-CDs for the Detection of Fe^2+^ and Fe^3+^

First, the solutions with different kinds of metal ions (Fe^2+^, Fe^3+^, Ca^2+^, Co^2+^, Cr^3+^, Cu^2+^, Hg^2+^, Mg^2+^, Mn^2+^, Pb^2+^ and Zn^2+^) were utilized to analyze the selectivity and interference of N, S-PPI-CDs for the detection of Fe^2+^ and Fe^3+^.

### 2.6. Fluorescence Sensitivity of N, S-PPI-CDs toward Fe^2+^, Fe^3+^ and LF

The changes in N, S-PPI-CDs’ fluorescence intensity were measured at 330 nm with increase in Fe^2+^ and Fe^3+^ concentration to investigate the behaviors of the fluorescence response. The changes in PPI-CDs’ fluorescence intensity were used as controls. Then, the fluorescence response behavior of N, S-PPI-CDs toward LF was studied by the similar approach.

## 3. Results and Discussion

### 3.1. FTIR of PPI-CDs and N, S-PPI-CDs

**Figure 1:**
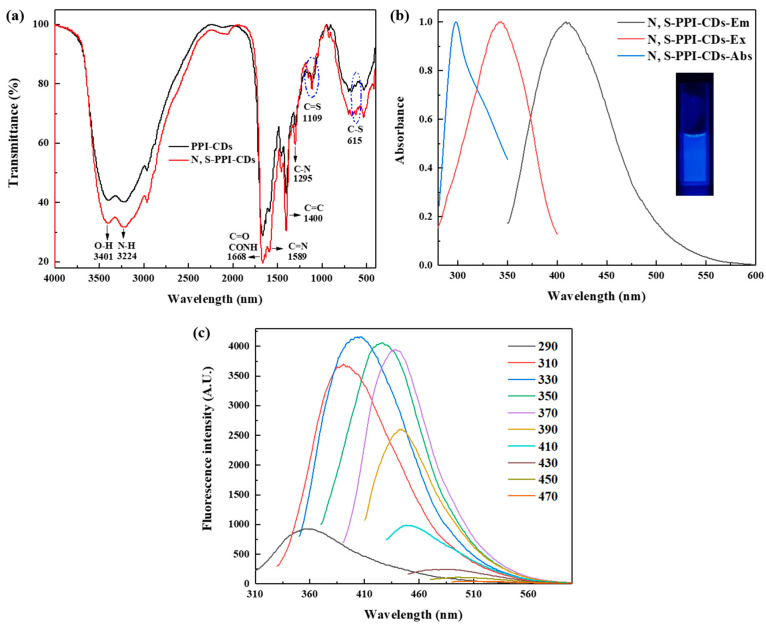
(**a**) FTIR spectrum of PPI-CDs and N, S-PPI-CDs; (**b**) UV–vis absorption spectrum and fluorescence spectrum of N, S-PPI-CDs (Inset: photograph of N, S-PPI-CDs solution under UV (365 nm) irradiation); (**c**) emission spectrum of N, S-PPI-CDs with the excitation wavelength increased from 290 to 470 nm at 20 nm intervals.

The PPI-CDs were prepared for comparison, to study the N, S-PPI-CDs. As shown in Figure 1a, the FT-IR spectra of the prepared PPI-CDs and N, S-PPI-CDs were studied to characterize the compositions. The FTIR spectra of the PPI-CDs and N, S-PPI-CDs showed similar peaks. The peaks at 3401 and 1668 cm^−1^ were confirmed to correspond to the stretching of O-H and C=O in COOH and CONH, respectively [29,30]. The peak at 3224 cm^−1^ was recognized as corresponding to the stretching vibration absorption of the N-H band [30]. Absorption bands peaking at 1589 and 1295 cm^−1^ originated from the other nitrogen-containing groups C=N and C-N, respectively [31]. In addition, sulfur-rich functional groups including C=S and C-S were located at 1109 and 615 cm^−1^, respectively [28]. Some characteristic absorption bands such as N-H (3224 cm^−1^), C=N (1589 cm^−1^), C-N (1295 cm^−1^), C=S (1109 cm^−1^) and C-S (615 cm^−1^) were stronger than those of the pure PPI-CDs, clearly indicating the groups of cysteamine has been bound to the surface of the N, S-PPI-CDs during the hydrothermal process.

### 3.2. Spectral Characteristics of N, S-PPI-CDs

The UV–vis absorption, excitation and emission spectra were measured to investigate the optical properties of the N, S-PPI-CDs. As shown in Figure 1b, the UV–vis absorption spectra of the as-prepared CDs showed an absorption peak centered at 296 nm, corresponding to the π–π* transition of carbon atoms [32]. The N, S-PPI-CDs illuminated blue fluorescence at 365 nm (UV excitation), while the maximum emission of the N, S-PPI-CDs was located at 404 nm with an excitation wavelength at 330 nm. To further study the fluorescence properties of the N, S-PPI-CDs, their emission strengths were measured at different excitation wavelengths. As shown in Figure 1c, when the excited wavelength increased from 290 nm to 470 nm, the emission peak redshifted gradually, and the fluorescence intensity decreased after reaching the maximum at 330 nm. The phenomenon was attributed to the excitation-dependent luminescent behavior of CDs. The fluorescence behavior has been widely described in other fluorescent carbon nanoparticles, which is attributed to the different sizes and surface emission sites of carbon dots [31,33,34].

### 3.3. XPS of PPI-CDs and N, S-PPI-CDs

**Figure 2:**
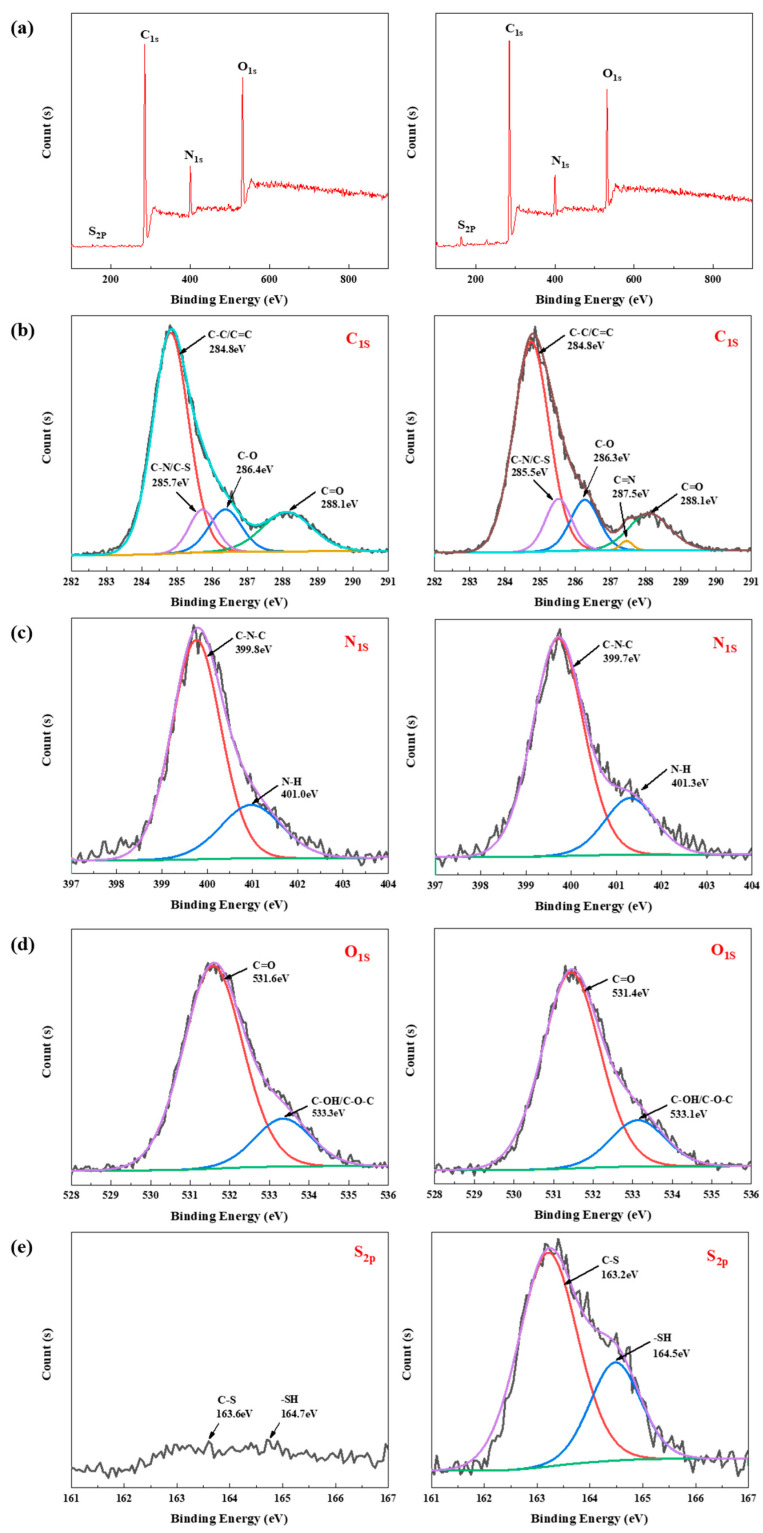
(**a**) XPS survey spectra of PPI-CDs and N, S-PPI-CDs; high-resolution XPS spectra of (**b**) C1s, (**c**) N1s, (**d**) O1s and (**e**) S2p of PPI-CDs and N, S-PPI-CDs, respectively.

The elemental composition and surface chemical states of the PPI-CDs and N, S-PPI-CDs were further compared by XPS. Figure 2a shows the full-scan XPS spectra of the PPI-CDs and N, S-PPI-CDs, with four peaks at 532.1, 400.2, 285.3 and 163.7 eV corresponding to O1s, N1s, C1s and S2p, respectively. The high-resolution spectra of C1s, N1s, O1s and S2p for the PPI-CDs and N, S-PPI-CDs are shown in Figure 2b–e, respectively, further analyzing their chemical composition and bonding [35]. As represented in Figure 2b, the C1s spectra of the PPI-CDs could be split into four peaks: C-C/C=C (284.8 eV), C-N (285.7 eV), C-O (286.4 eV) and C=O (288.1 eV). In addition, the N, S-PPI-CDs showed five peaks: C-C/C=C (284.8 eV), C-N/C-S (285.5 eV), C-O (286.2 eV), C=N (287.5 eV) and C=O (288.1 eV) [34,36,37]. The high-resolution N1s and O1s XPS spectra of the PPI-CDs or N, S-PPI-CDs clearly showed two peaks, respectively, which represent oxygen bonds (C=O, C-OH/C-O-C) and nitrogen bonds (C-N-C, N-H) [34,38]. The percentage of chemical bonds N-H and C=N in the N, S-PPI-CDs were 3.09% and 2.01%, respectively, while those in the PPI-CDs were 2.49% and 0%, respectively. This indicates that the content of chemical bonds N-H and C=N in the N, S-PPI-CDs evidently increased, in comparison to that of PPI-CDs, which was consistent with the FTIR results. As observed in Figure 2e, the S2p spectra of the N, S-PPI-CDs contained distinct peaks of C-S (163.2 eV) and -SH (164.5 eV) compared with those of the PPI-CDs [39]. This further manifested that S atoms have been resoundingly doped into the N, S-PPI-CDs. Therefore, the surface of the N, S-PPI-CDs is enriched with −COOH, −OH, −NH_2_ and −SH compared to the N, S-CDs and PPI-CDs, which may make it have a more outstanding advantage in providing active sites.

### 3.4. Biocompatibility of PPI-CDs and N, S-PPI-CDs

**Figure 3:**
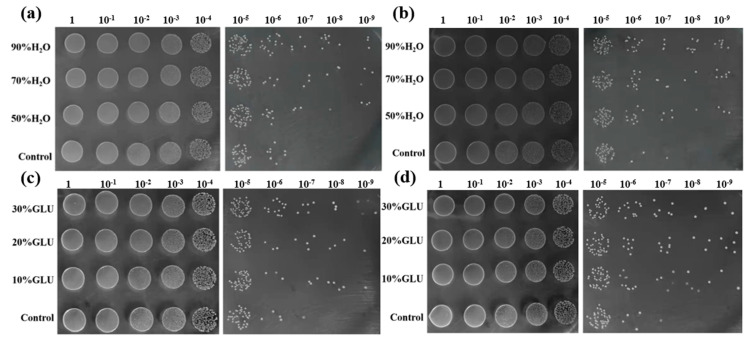
Viable count of *Lactobacillus plantarum* after (**a**) PPI-CDs or (**b**) N, S-PPI-CDs solution (0.01 g/mL) replaced partial water, and (**c**) PPI-CDs or (**d**) N, S-PPI-CDs replaced partial glucose (GLU) (the dilution ratio of bacterial solution is 1→10^9^).

The non-toxic nature of carbon dots makes them a great potential in the field of biological sample detection. To determine the nontoxicity of the as-synthesized CDs, we studied the influence of the PPI-CDs and N, S-PPI-CDs’ partial substitution for H_2_O or GLU upon the growth of *Lactobacillus plantarum*. As illustrated in Figure 3, the number of viable bacteria evidently increased with the partial replacement of the PPI-CDs and N, S-PPI-CDs for H_2_O or GLU and showed an upward trend with the raise of substitution rate. This indicates that the two as-synthesized CDs are good bacterial carbon sources. Interestingly, N, S-PPI-CDs substitution is more beneficial to the growth of *Lactobacillus plantarum* compared with PPI-CDs substitution, which can be attributed to the successful doping of groups from cysteamine in the carbon dots that further promotes the growth of *Lactobacillus plantarum*. These results demonstrated the N, S-PPI-CDs are nontoxic, and this kind of new carbon dots has great potential application as a new culture medium.

### 3.5. Morphology Characterization of CDs

**Figure 4:**
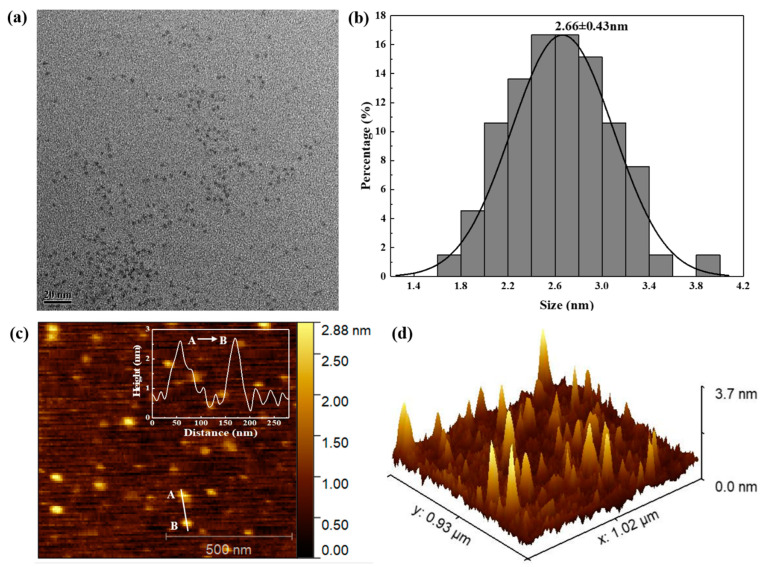
Morphology characterization of the N, S-PPI-CDs CDs. (**a**) TEM of N, S-PPI-CDs CDs; (**b**) statistical distribution of the sizes of the N, S-PPI-CDs; (**c**) AFM image of N, S-PPI-CDs (Inset: height distribution of N, S-PPI-CDs); (**d**) three-dimensional AFM image of the N, S-PPI-CDs.

The morphology characteristics of the N, S-PPI-CDs were studied by TEM and AFM. As shown in Figure 4a, the as-prepared CDs were well-dispersed and had a monodispersed spherical shape. The statistical chart with a uniform size distribution of the N, S-PPI-CDs was measured according to a TEM picture (Figure 4b). The sizes of the N, S-PPI-CDs varied from 1.6 to 4.0 nm, with a diameter of 2.66 ± 0.43 nm. The AFM picture of the N, S-PPI-CDs in Figure 4c further clarifies the morphology of the CDs. The height profile curve (inset in Figure 4c) indicates that the average height of the N, S-PPI-CDs was 2.7 ± 0.1 nm, which is consistent with the TEM measurement results. In addition, the three-dimensional height distribution of the N, S-PPI-CDs is depicted in Figure 4d. This reveals that the height of the N, S-PPI-CDs was well-distributed. Combining the above morphology characterizations, we can confirm that the N, S-PPI-CDs were homogeneous spheres with an average range of 2–3 nm.

### 3.6. Fluorescent Selectivity and Interference of N, S-PPI-CDs for the Detection of Fe^2+^ and Fe^3+^

**Figure 5:**
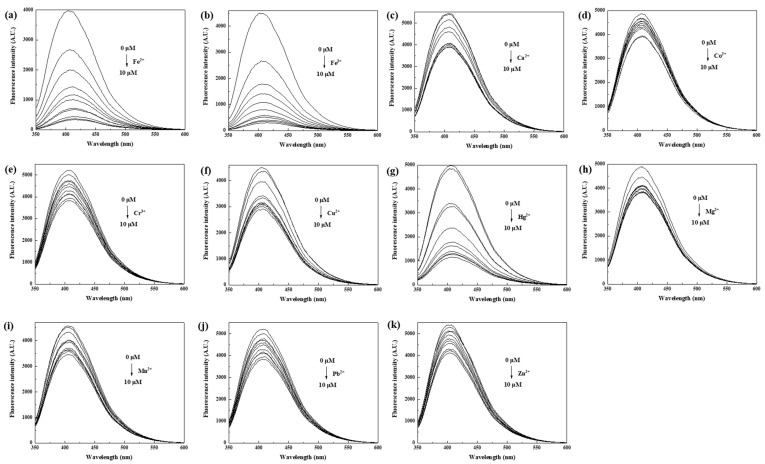
The variation in fluorescence intensity of N, S-PPI-CDs (1 g/L) with the increase in (**a**) Fe^2+^, (**b**) Fe^3+^, (**c**) Ca^2+^, (**d**) Co^2+^, (**e**) Cr^3+^, (**f**) Cu^2+^, (**g**) Hg^2+^, (**h**) Mg^2+^, (**i**) Mn^2+^, (**j**) Pb^2+^ and (**k**) Zn^2+^ concentration.

**Figure 6:**
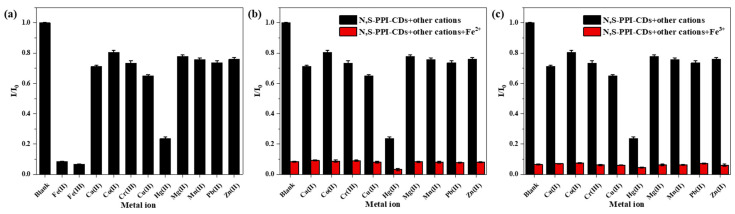
(**a**) The selectivity of the N, S-PPI-CDs (1 g/L) for the detection of metal ions (10 μM); the interference of the N, S-PPI-CDs (1 g/L) for the detection of (**b**) Fe^2+^ or (**c**) Fe^3+^. The concentration of Fe^2+^ or Fe^3+^ was 10 μM, and other cations’ concentrations were the same as Fe^2+^ or Fe^3+^.

It has been reported that functional groups on the surface of CDs can interact with metal ions in coordination, resulting in changes in fluorescence intensity [40]. To investigate the selectivity of the N, S-PPI-CDs to metal ions, fluorescence quenching was examined using 11 different metal ions (Fe^2+^, Fe^3+^, Ca^2+^, Co^2+^, Cr^3+^, Cu^2+^, Hg^2+^, Mg^2+^, Mn^2+^, Pb^2+^ and Zn^2+^) with 0–10 μM (Figure 5a–k). It was observed that the fluorescence intensity decreased with an increase in metal ions. In addition, the fluorescence intensity for Fe^2+^, Fe^3+^ and Hg^2+^ decreased most significantly. Moreover, the *I/I_0_* was analyzed to characterize the fluorescent selectivity and sensitivity of CDs to metal ions. The fluorescent intensity of CDs without the addition of metal ions is expressed as *I_0_*, and the fluorescence intensity of the CDs solution after the addition of metal ions is expressed as *I*. As displayed in Figure 6a, Fe^2+^, Fe^3+^ and Hg^2+^ evoked prominent decreases in the fluorescence of the N, S-PPI-CDs solution compared with other metal ions, which indicates that the N, S-PPI-CDs have an excellent selectivity toward Fe^2+^, Fe^3+^ and Hg^2+^. As depicted in Figure 6b,c, the coexistence of the Hg^2+^ ion induced a marked decrease in the fluorescence intensity. Other competitive ions would not cause obvious interference to the selective sensing of Fe^2+^ or Fe^3+^.

According to the CODEX STAN 193-1995 Codex General Standard For Contaminants And Toxins In Food And Feed issued by the Codex Alimentarius Commission (CAC), mercury content in natural mineral water and food grade salt is limited to 0.001 and 0.1 mg/kg, respectively. In addition, the GB 2762-2017 National Food Safety Standard Limit of Pollutants in Food established maximum limits for mercury in other food categories, none of which exceed 0.1 mg/kg. As shown in Figure 5g, when the Hg^2+^ concentration was 0–1 μM (> 0.1 mg/kg), the fluorescence intensity of the N, S-PPI-CDs was basically unchanged, indicating that the N, S-PPI-CDs have no selectivity to Hg^2+^. Therefore, the N, S-PPI-CDs can avoid the interference of Hg^2+^ in the selective detection of Fe^2+^ and Fe^3+^ in food.

### 3.7. Fluorescent Sensitivity of N, S-PPI-CDs toward Fe^2+^ or Fe^3+^

**Figure 7:**
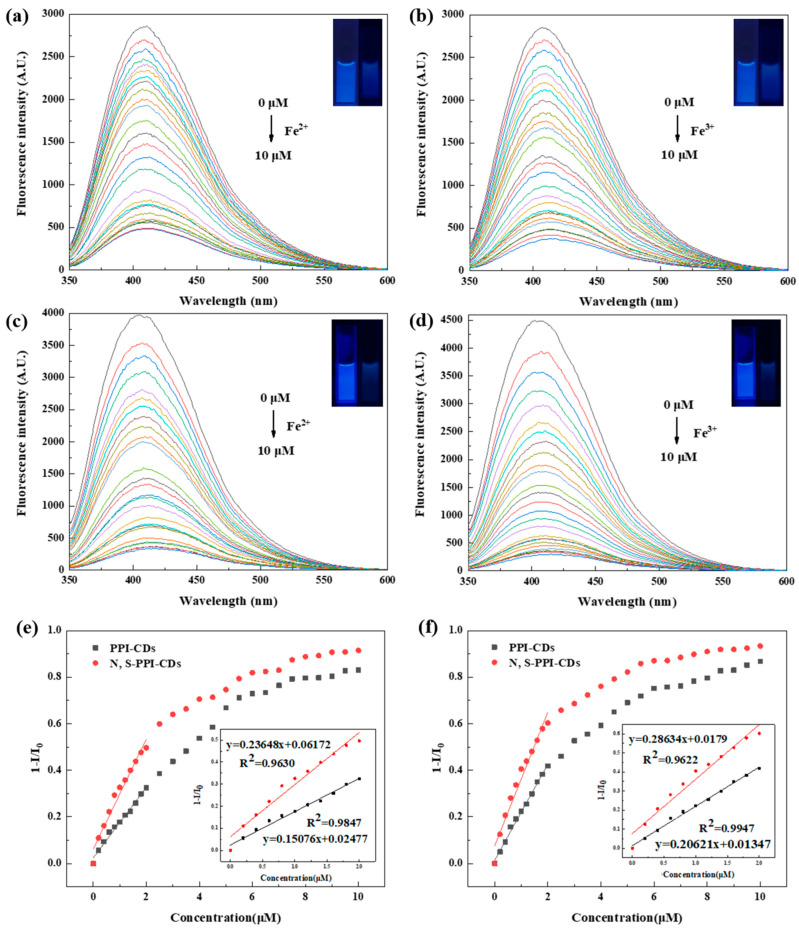
Fluorescence response of PPI-CDs with different concentrations of (**a**) Fe^2+^ and (**b**) Fe^3+^ (Inset: photographs of PPI-CDs solution under UV (365 nm) irradiation after adding Fe^2+^ or Fe^3+^ (0 and 10 μM), respectively); fluorescence response of N, S-PPI-CDs with different concentrations of (**c**) Fe^2+^ and (**d**) Fe^3+^ (Inset: photographs of N, S-PPI-CDs solution under UV (365 nm) irradiation after adding Fe^2+^ or Fe^3+^ (0, 10 μM), respectively); Stern–Volmer plots of PPI-CDs and N, S-PPI-CDs toward (**e**) Fe^2+^ and (**f**) Fe^3+^, respectively.

To further assess the sensitivity of the as-prepared CDs toward Fe^2+^ and Fe^3+^, we measured the fluorescent intensity of the PPI-CDs and N, S-PPI-CDs as the concentration of Fe^2+^ and Fe^3+^ increased, respectively. As shown in Figure 7c,d, with the continuous addition of Fe^2+^ or Fe^3+^, a more obvious decrease in the fluorescence intensity of the N, S-PPI-CDs was observed without wavelength changes, compared to that of the PPI-CDs (Figure 7a,b). When the added concentration of Fe^2+^ reaches 10 μM, the quenching rate of N, S-PPI-CDs can reach 92%. Meanwhile, the quenching rate of N, S-PPI-CDs can reach 94% when the added concentration of Fe^3+^ reaches 10 μM. In addition, we analyzed the Stern–Volmer plots with quenching efficiency as the Y-axis and Fe^2+^ and Fe^3+^ concentration as the X-axis. As depicted in Figure 7e,f, the plots of the PPI-CDs and N, S-PPI-CDs exhibit a rising trend with an increase in the Fe^2+^ or Fe^3+^ concentration in the range of 0–10 μM and presented a good linear relationship under a low concentration (0–2 μM). In addition, the slope of the N, S-PPI-CDs plot was obviously higher than that of the PPI-CDs plot, which means the N, S-PPI-CDs have great potential in Fe^2+^ or Fe^3+^ fluorescent-sensing detection with a low concentration. This correlation can be formulated with Equation (1) [41]:

(1) $$1-\frac{I}{I_{0}}=K_{\mathrm{sv}}\left[M\right]$$

The fluorescent intensity of CDs with and without the addition of Fe^2+^ or Fe^3+^ are expressed as I or I_0_, respectively; The calculated slope of the Stern–Volmer plot is expressed as K_sv_ (quenching constant); The concentration of Fe^2+^ or Fe^3+^ is expressed as [M].

To evaluate the sensitivity of the N, S-PPI-CDs to detect Fe^2+^ or Fe^3+^, we calculated their limit of detection (LoD) according to Equation (2). The intensity standard deviation of the blank sample for 10 measurements is expressed as S_d_ [42,43]. The LoD of the N, S-PPI-CDs toward Fe^2+^ and Fe^3+^ was determined to be 0.21 μM and 0.17 μM, respectively. The obtained data were significantly lower than the limit of iron in drinking water (~5.537 μM) set by the US Environmental Protection Agency (EPA) [31]. This indicates that the N, S-PPI-CDs can be used for the quantitative detection of Fe^2+^ or Fe^3+^ in an aqueous solution.

(2) $$\mathrm{LoD}=\frac{3S_{d}}{K_{\mathrm{sv}}}$$

In order to assess the potential of N, S-PPI-CDs as a fluorescent probe to detect Fe^2+^ or Fe^3+^, a comparison of the previous literature on CDs-based Fe^2+^ or Fe^3+^ assays is summarized in Table 1. It turns out that the LoD obtained in this work is apparently lower than in other relevant reports. This indicates that the N, S-PPI-CDs are a great potential probe for Fe^2+^ and Fe^3+^ detection.

### 3.8. Fluorescent-Sensing Performance of LF

**Figure 8:**
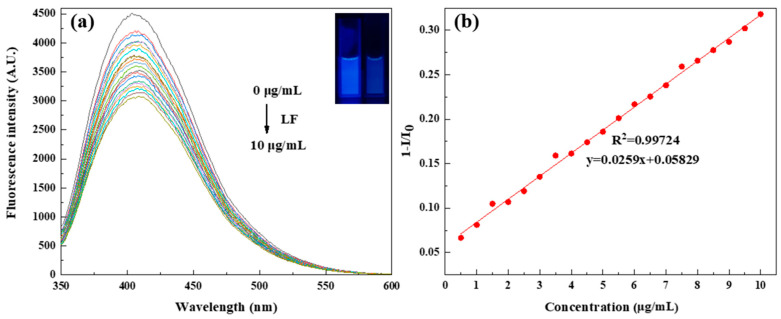
(**a**) PL response of N, S-PPI-CDs with different concentrations of lactoferrin (LF) (Inset: photographs of N, S-PPI-CDs solution under UV (365 nm) irradiation after adding LF (0 and 10 μg/mL), respectively.); (**b**) Stern–Volmer plot of N, S-PPI-CDs toward LF.

LF is a multifunctional protein with varying activities: promoting intestinal iron absorption, anti-inflammation, antibacterial, etc. [17,18]. This protein is an iron-binding glycoprotein with two lobes, which can reversibly bind to Fe^2+^/Fe^3+^ [13]. Therefore, the content of LF can be detected by using the fluorescence-intensity changes of the fluorescent compounds with a strong binding ability to Fe^2+^/Fe^3+^. We measured the fluorescence-emission variation of the N, S-PPI-CDs solution with an increase in the LF concentration. As shown in Figure 8a, with the continuous addition of LF, a gradual decrease in the fluorescence intensity of the N, S-PPI-CDs was observed. When the LF concentration increased from 0.5 μg/mL to 10 μg/mL, it showed a good linear relationship (Figure 8b). The linear regression equation was y = 0.0259x + 0.05829 (R^2^= 0.99724). In addition, the calculated LoD value was 1.92 µg/mL, which was lower than the values of other assays (Table 2). This suggests the as-synthesized N, S-PPI-CDs have promising potential as fluorescent sensors applied for LF detection.

Based on the above-mentioned results, Fe^2+^/Fe^3+^ can specifically quench the fluorescence of the as-synthesized N, S-PPI-CDs (Scheme 1). This can be attributed to the fact that Fe^2+^/Fe^3+^ have a stronger affinity toward the functional groups on the N, S-PPI-CDs surface (including hydroxyl, carboxyl and amino) than other metal ions [45,57,58]. In addition, the potential strong coordination interaction between the doped S element and the iron ion is of great significance to the quenching of the N, S-PPI-CDs’ fluorescence [59]. Due to Fe^2+^/Fe^3+^ being contained in LF, we can realize the detection of LF with the specific capture of Fe^2+^/Fe^3+^ in LF by the N, S-PPI-CDs. This shows a good linear relationship between the quenching efficiency of the N, S-PPI-CDs and the LF concentration. Therefore, the LF concentration could be determined according to the degree of fluorescence quenching of the N, S-PPI-CDs solution.

### 3.9. Application of N, S-PPI-CDs in Lactoferrin Supplements

To verify the practical applicability of the N, S-PPI-CDs for the detection of lactoferrin in real samples, the well-received lactoferrin supplements were selected for analysis in this work. The lactoferrin content obtained by fluorescence detection based on the N, S-PPI-CDs was compared with the standard content, as shown in Table 3. The recovery of LF in lactoferrin supplementation floated in the range of 99.42–102.48%, with a relative standard deviation (RSD) of less than 1.00%, which confirmed the feasibility of the method. Therefore, the fluorescence sensor based on the N, S-PPI-CDs is an ideal sensor that can achieve a simple, sensitive and rapid determination of lactoferrin in immunity supplements.

## 4. Conclusions

In this work, we developed a simple, efficient and economical fluorescent N, S-PPI-CDs nanosensor for LF detection, which is prepared by the hydrothermal synthesis of carbon dots using a protein (PPI) and cysteamine as precursors. A toxicity experiment indicated that the N, S-PPI-CDs were conducive to *Lactobacillus plantarum* growth, which verified their excellent biocompatibility. Meanwhile, the CDs were uniformly dispersed spheres with a diameter of only 2–3 nm, which would provide an abundance of active binding sites. The PL of the N, S-PPI-CDs can be obviously quenched by Fe^2+^, Fe^3+^ and Hg^2+^ compared to other metal ions. However, the maximum limit for the mercury in food set by the CAC and GB does not exceed 0.1 mg/kg (<1 μM), which hardly caused any fluorescence quenching of the N, S-PPI-CDs. This suggests that the N, S-PPI-CDs can be used as a highly selective fluorescent-detection probe for Fe^2+^/Fe^3+^ in food without interference from Hg^2+^. In addition, the LoD can be as low as 0.21 μM/0.17 μM. Furthermore, due to the principle that Fe^2+^/Fe^3+^ can reversibly bind to LF, the N, S-PPI-CDs were used to detect LF and showed a high sensitivity for LF, with an LoD of 1.92 µg/mL. Moreover, this method was further used to assay LF in LF supplements, with recoveries ranging from 99.42–102.48%. The successful quantification of LF demonstrated the application potential of a nanofluorescent N, S-PPI-CDs probe in real samples.

## Figures and Tables

**Table 1:** Fluorescence sensors in the literature for Fe^3+^/Fe^2+^ detection.

Sensor System	Analyte	Linear Range (μM)	Limit of Detection (LOD) (μM)	Reference
N-doped carbon dots	Fe^3+^	10–100	32	[44]
S, N, Co-doped carbon dots	Fe^3+^ and Fe^2+^	5–200 and 5–300	2.86 and 2.06	[23]
N, P-doped carbon quantum dots	Fe^3+^ and Fe^2+^	0–250 and 0–600	0.447 and 0.298	[45]
Nitrogen-doped CDs	Fe^2+^	0–500	0.702	[46]
Carbon quantum dots	Fe^3+^	50–650	0.196	[47]
Carbon-dot-decorated natural microcline	Fe^3+^	0–30	61.6	[48]
Polyurethane–nitrogen-doped carbon dot nanocomposites	Fe^3+^	10–250	10.10	[49]
Dual-emissive fluorescent probe synthesized using Eu^3+^ and 2-hydroxyterephthalic acid	Fe^2+^	0–50	0.32	[50]
Spiropyran-based fluorescent probe	Fe^2+^	4–20	0.77	[51]
Eu^3+^-doped metal–organic framework	Fe^3+^	0–6	0.897	[52]
N, S-PPI-CDs	Fe^3+^ & Fe^2+^	0–2	0.17 & 0.21	this work

**Table 2:** Different detect methods in the literature for LF detection.

Detect Method	Analyte	Limit of Detection (LoD)	Reference
Colorimetric	LF	110 µg/mL	[53]
Aptamer affinity column	LF	3 µg/mL	[2]
Reversed-phase high-performance liquid chromatography	LF	35.46 µg/mL	[10]
Thin-layer chromatography	LF	3.5 µg/mL	[3]
HPLC-UV	LF	0.6 mg/100 g (liquid samples) 3 mg/100 g (solid samples)	[54]
LC−MS/MS	LF	0.3 mg/100 g	[11]
Ultrahigh-performance liquid chromatography–tandem mass spectrometry	LF	3.8 mg/kg	[55]
Ultrahigh-performance liquid	LF	1 mg/100 g	[56]
N, S-PPI-CDs	LF	1.92 µg/mL	this work

**Table 3:** Determination of LF in real lactoferrin samples.

Sample	Standard (mg/mL)	Found (mg/mL)	Recovery (%)	RSD (%)
Sample 1	10.00	10.24	102.48	1.00
Sample 2	25.80	25.65	99.42	0.86

## Data Availability

All data generated or analyzed during this study are included in this article.
